# Association of Familial Hypercholesterolemia and Statin Use With Risk of Dementia in Norway

**DOI:** 10.1001/jamanetworkopen.2022.7715

**Published:** 2022-04-19

**Authors:** Liv J. Mundal, Jannicke Igland, Karianne Svendsen, Kirsten B. Holven, Trond P. Leren, Kjetil Retterstøl

**Affiliations:** 1 The Lipid Clinic, Department of Endocrinology, Morbid Obesity, and Preventive Medicine, Oslo University Hospital, Oslo, Norway; 2 Department of Global Public Health and Primary Care, University of Bergen, Bergen, Norway; 3 Department of Health and Social Sciences, Institute of Health and Caring Science, Western Norway University of Applied Sciences, Bergen, Norway; 4 Department of Nutrition, Institute of Basic Medical Sciences, Faculty of Medicine, University of Oslo, Oslo, Norway; 5 National Advisory Unit on Familial Hypercholesterolemia, Department of Endocrinology, Morbid Obesity, and Preventive Medicine, Oslo University Hospital, Oslo, Norway; 6 Unit for Cardiac and Cardiovascular Genetics, Oslo University Hospital, Oslo, Norway

## Abstract

**Importance:**

Hypercholesterolemia, which is a cardiovascular risk factor, may also be associated with dementia risk. The benefit of statin treatment on dementia risk is controversial.

**Objective:**

To determine whether individuals with familial hypercholesterolemia (FH), who have been exposed to lifelong hypercholesterolemia, have an excess risk of dementia and whether statin use is associated with dementia risk.

**Design, Setting, and Participants:**

This was a prospective cohort study performed from 2008 to 2018 in Norway. Statistical analysis was performed from January 2021 to February 2022. This study included individuals with genetically verified FH and age-matched and sex-matched controls obtained from the general Norwegian population.

**Exposures:**

Dementia was defined according to *International Statistical Classification of Diseases and Related Health Problems, Tenth Revision* codes F00-03 and G30.

**Main Outcomes and Measures:**

Incident cases of total dementia, vascular dementia, Alzheimer disease–dementia in Alzheimer disease, and data on lipid-lowering medication were obtained from the Norwegian Patient Registry, Cause of Death Registry, and the Norwegian Prescription Database. Hazard ratios (HRs) for risk of dementia for individuals with FH vs matched controls were calculated using Cox regression. The cumulative sum of defined daily doses (DDDs) of statins prescribed during study follow-up was calculated for individuals with FH and was analyzed as a time-varying covariate with 3 levels: 1 to 4999 DDDs, 5000 to 10 000 DDDs, and more than 10 000 DDDs.

**Results:**

Among the 3520 individuals with FH (1863 women [52.9%]; mean [SD] age at the start of follow-up, 51.8 [11.5] years) and the 69 713 controls (36 958 women [53.0%]; mean [SD] age at the start of follow-up, 51.7 [11.5] years), 62 patients with FH (39 women [62.9%]) and 1294 controls (801 women [61.9%]) had developed dementia over the course of 10 years of follow-up. Most dementia cases occurred among individuals aged 70 years and older (39 patients with FH [62.9%] and 870 patients [67.2%] in the control group). We found no excess risk of dementia in patients with FH vs matched controls (HR for total dementia, 0.9; 95% CI, 0.7-1.2). There was no association between cumulative DDDs of statins and total dementia in patients with FH with HRs of 1.2 (95% CI, 0.4-3.8) for cumulative DDDs of 5000 to 10 000 and 1.9 (95% CI, 0.7-5.0) for cumulative DDDs greater than 10 000.

**Conclusions and Relevance:**

These findings suggest that individuals with FH have no excess risk of dementia compared with age-matched and sex-matched controls and that there is no association between use of statins and risk of dementia in patients with FH.

## Key Points

**Question:**

Is genetically caused high low-density lipoprotein cholesterol associated with increased risk of dementia?

**Findings:**

This cohort study including 3520 individuals with genetically verified familial hypercholesterolemia (FH) and 69 713 age-matched and sex-matched controls found no excess risk of dementia for patients with FH vs matched controls. Furthermore, there was no association between statin use and dementia.

**Meaning:**

These findings suggest that individuals with FH have no excess risk of dementia and that there is no association between long-term high-intensity statin use and dementia risk.

## Teaser

This cohort study examines whether individuals with familial hypercholesterolemia have an excess risk of dementia compared with controls and whether long-term statin use is associated with dementia risk.

## Introduction

Dementia is one of the major causes of disability worldwide, affecting approximately 50 million people, with nearly 10 million new cases yearly.^1^ There are many different forms of dementia, of which Alzheimer disease (AD) is the most common, accounting for 60% to 80% of all cases.^1,2,3,4^ It is estimated that by 2050, 1 in 85 persons worldwide will have dementia.^5^ The incidence of AD is approximately 13% among individuals aged 65 years and older and 45% among individuals aged 85 years and older.^6^ As the world’s population ages, we are facing a global epidemic of AD.^5^ A delay in the onset of dementia by 2 years could potentially lower the prevalence of AD by more than 22 million cases over the next 40 years.^7^

Vascular dementia is the second most common form of dementia, accounting for approximately 20% of all cases, followed by Lewy body dementia (approximately 15% of cases), frontotemporal dementia (approximately 8%-10% of cases), and other forms of dementia (1% of cases).^2,3^ Different forms of dementia often coexist.^1^

Cardiovascular disease (CVD) risk factors are important in the development of dementia.^8^ One-third of all dementia cases may be associated with CVD risk factors and may, therefore, be preventable.^8^ There is a positive association between levels of low-density lipoprotein cholesterol (LDL-C) and dementia risk.^9^ A high LDL-C level may be associated with AD neurodegeneration.^4^ Statins may slow the conversion from mild cognitive impairment (MCI) to dementia.^4^ Recent research suggests a possible link between inflammation, dyslipidemia, atherosclerosis, and dementia.^7^

Familial hypercholesterolemia (FH) may represent a model disease for studying atherosclerosis and the risk of dementia. FH is an inherited disease caused by a mutation in the LDL-C receptor gene.^10^ A recent meta-analysis^11^ reported a prevalence of FH in the general population of 1 in 313 individuals. FH leads to high levels of plasma LDL-C from birth and excess risk of early atherosclerosis and premature CVD.^12^

The aim of the current study was to compare the incidence of dementia, including total dementia, vascular dementia, and AD–dementia in AD, between patients with genetically verified FH and an age-matched and sex-matched control cohort randomly selected from the Norwegian population. A secondary aim was to study the association between use of lipid-lowering treatment and the risk of developing dementia among persons with FH.

## Methods

### Approvals

This study was approved by the Regional Committee of Medical and Health Research Ethics South-Eastern Norway. The study complied with the Declaration of Helsinki^13^ and was reported to the Norwegian Data Protection Official at Oslo University Hospital. Written informed consent is required to be included in the FH cohort, and individuals within the FH cohort had the opportunity to withdraw from the study. No consent was needed to be included in the control cohort because the data were anonymized.

### Study Design

This was a registry-based, prospective cohort study including individuals with genetically verified FH and age-matched and sex-matched controls obtained from the general Norwegian population during 2008 to 2018. This study followed the Strengthening the Reporting of Observational Studies in Epidemiology (STROBE) reporting guideline.

### Registries and Data Collection

All patients with genetically verified FH in Norway are included in the Unit for Cardiac and Cardiovascular Genetics (UCCG) registry at Oslo University Hospital.^14^ The UCCG registry, the FH cohort, and the control group matched on gender and birth year have been described elsewhere.^14,15,16^ In the current study, we included patients with FH who were genetically diagnosed during 1992 to 2014 and their matched controls. The controls were matched on age and sex to the FH population in a 1:20 ratio. A flowchart for definition of the study population is given in the eFigure in the Supplement.

Data on occurrence of dementia were obtained through linkage with the Norwegian Patient Registry (NPR),^17^ for which the Norwegian Directorate of Health is responsible, and the Norwegian Cause of Death Registry (NCoDR)^18^ by using each individual’s unique personal identification number. From NPR, which contains patient data from 2008 and onward, we obtained data on all contacts (hospitalizations or outpatient visits) with dementia reported as primary or secondary diagnostic codes at somatic hospitals, contracted private specialists, rehabilitation facilities, and psychiatric institutions for the period 2008 to 2018. Data from NCoDR included information on date of death and the underlying, immediate, or contributing cause of death. An incident case of dementia was defined as the first time occurrence with dementia as primary or secondary diagnosis in NPR or as the underlying cause of death without any previously reported dementia diagnostic codes. Dementia was defined as total dementia (*International Statistical Classification of Diseases and Related Health Problems, Tenth Revision *[*ICD-10*] codes F00-03 and G30), vascular dementia (*ICD-10* code F01), and AD–dementia in AD (*ICD-10* codes G30 and F00).

The start of follow-up was defined as the latest date of the following 3 dates: the registration date in the UCCG registry for the FH diagnosis, January 1 in the year the person reached age 40 years, or January 1, 2008. The control persons within each matched set had the same date for start of follow-up as the FH person in the set. Follow-up time was calculated from the start of follow-up until the end point, death from other causes, or December 31, 2018 (whichever occurred first). January 1, 2008, was chosen as the earliest possible start of follow-up because we did not have data on dementia diagnosis before this date.

To evaluate the use of lipid-lowering drugs, the UCCG registry was linked to the Norwegian Prescription Database (NorPD).^19^ NorPD contains data on prescription of pharmaceuticals from 2004 and onward. We included data on all dispensed prescriptions of statins with oral formulation with Anatomical Therapeutic Chemical Classification System (ATC) codes starting with C10AA from 2004 until the end of follow-up. For each prescription of statins, information on the amount of drug prescribed was reported in milligrams and as the number of defined daily doses (DDDs), as endorsed by the World Health Organization, where DDD is the assumed daily average maintenance dose for a drug used for its main indication in adults.^20^ Because the type of statins prescribed varied between persons and also within persons over time, we used DDD to obtain a standardized measurement unit that can be compared across types of statins.

### Statistical Analysis

Descriptive data are presented as means with SDs for normally distributed continuous variables and as frequencies (percentages) for categorical variables. Differences between FH and controls in baseline characteristics were tested using 2-sided *t* test for age and 2-sided χ^2^ test for categorical variables. Crude incidence rates per 1000 person-years of total dementia, vascular dementia, and AD–dementia in AD during 2008 to 2018 were calculated separately for patients with FH and controls.

Relative differences in the risk of dementia between patients with FH and control participants were estimated as hazard ratios (HRs) with 95% CIs from Cox regression with adjustment for sex and age at the start of follow-up. Our aim was to estimate the total association of FH with dementia risk; therefore, we did not adjust for variables that could be intermediate factors, such as previous CVD. In addition, we also did analyses stratified by sex and age group at the start of follow-up. Because patients with FH have high risk of mortality from coronary heart disease, we performed sensitivity analyses using Fine and Gray competing risk regression, with death from other causes than dementia treated as competing events.^21^ The cumulative incidence of total dementia according to sex and age in patients with FH vs matched controls were calculated and displayed graphically.

The association between use of statins and risk of total dementia in patients with FH was analyzed using Cox regression, with cumulative DDDs of statins as a time-varying covariate. We restricted the analyses to persons with FH who had at least 1 statin prescription during follow-up. The follow-up time for each person was split into 3 intervals, with cutoffs at 5000 and 10 000 DDDs, resulting in a categorical time-varying covariate with 3 levels: 1 to 4999 DDDs, 5000 to 10 000 DDDs, and more than 10 000 DDDs. Age was used as the time scale in the analyses to take into account the association between statin use and age, and all models were adjusted for sex. In additional models, we also adjusted for the following covariates: coronary heart disease (*ICD-10* codes I20-I25), hypertension (*ICD-10* codes I10-I15), atrial fibrillation (*ICD-10* code I48), stroke (*ICD-10* codes I6I, I63 [-I63.6], I64), antihypertensive drugs (ATC-group C01), antithrombotic drugs (ATC-group B01A), and diabetes-related drugs (ATC-group A10) at the start of follow-up. Use of pharmaceuticals was defined as at least 2 dispensed prescriptions the same year as start of follow-up.

Statistical analysis was performed from January 2021 to February 2022. The level of statistical significance was set at *P* < .05 in all analyses. Stata statistical software version 16 (StataCorp) was used in all analyses.

## Results

The baseline characteristics of the study population, including 3520 individuals with genetically verified FH and 69 713 age-matched and sex-matched controls, are shown in Table 1. Women accounted for 53.0% of the study population (1863 women [52.9%] with FH and 36 958 women [53.0%] in the control group). The mean (SD) age at the start of follow-up was 51.8 (11.5) years in the FH group and 51.7 (11.5) years in the control group; the mean (SD) age at diagnosis was 45.1 (14.0) years for patients with FH. Significantly more individuals with FH had prior coronary heart disease and hypertension compared with controls and, accordingly, higher use of antithrombotic and antihypertensive drugs (Table 1). At the start of follow-up, 1404 women with FH (75.4%) and 1364 men with FH (82.3%) were taking statin treatment (*P* < .001 for sex difference; χ^2^_1_ = 25.3).

**Table 1.** Baseline Characteristics of Patients With FH and Age-Matched and Sex-Matched Controls

Characteristic	Patients, No. (%)		*P* value
	FH (n = 3520)	Controls (n = 69 713)	
Sex			
Male	1657 (47.1)	32 755 (47.0)	.92
Female	1863 (52.9)	36 958 (53.0)	
Age at time of FH diagnosis, mean (SD), y	45.1 (14.0)	Not applicable	.90
Age at start of follow-up, mean (SD), y	51.8 (11.5)	51.7 (11.5)	
Age group at start of follow-up, y			
40-59	2607 (74.1)	51 669 (74.1)	.98
60-69	584 (16.6)	11 603 (16.6)	
≥70	329 (9.4)	6441 (9.2)	
Comorbidities before start of follow-up^a^			
Coronary heart disease	221 (6.3)	677 (1.0)	<.001
Stroke	8 (0.2)	117 (0.2)	.41
Atrial fibrillation	26 (0.7)	340 (0.5)	.04
Hypertension	82 (2.3)	1090 (1.6)	<.001
Medications at start of follow-up^b^			
Antithrombotics	1077 (54.3)	6272 (28.3)	<.001
Antihypertensives	149 (7.5)	906 (4.1)	<.001
Diabetes-related drugs	131 (6.4)	2370 (9.5)	<.001
Statin use at start of follow-up, by age group^c^			
All ages	2768 (78.6)	7007 (10.1)	<.001
40-59 y	1939 (74.4)	2298 (4.5)	<.001
60-69 y	535 (91.6)	2635 (22.7)	<.001
≥70 y	294 (89.4)	2074 (32.2)	<.001

Abbreviation: FH, familial hypercholesterolemia.

^a^ *International Statistical Classification of Diseases and Related Health Problems, Tenth Revision* codes for comorbidities are as follows: coronary heart disease, codes I20 to I25; stroke, codes I61, I63 (-I63.6), and I64; atrial fibrillation, code I48; and hypertension, codes I10 to I15.

^b^ For antithrombotics, patients had to have at least 2 prescriptions of drugs within Anatomical Therapeutic Chemical Classification System (ATC) group B01A the same year as start of follow-up. For antihypertensives, patients had to have at least 2 prescriptions of drugs within ATC group C01 the same year as start of follow-up. For diabetes-related drugs, patients had to have at least 2 prescriptions of drugs within ATC group A10 the same year as start of follow-up.

^c^ For statins, patients had to have at least 2 prescriptions of ATC group C10AA the same year as start of follow-up.

**Figure 1.**
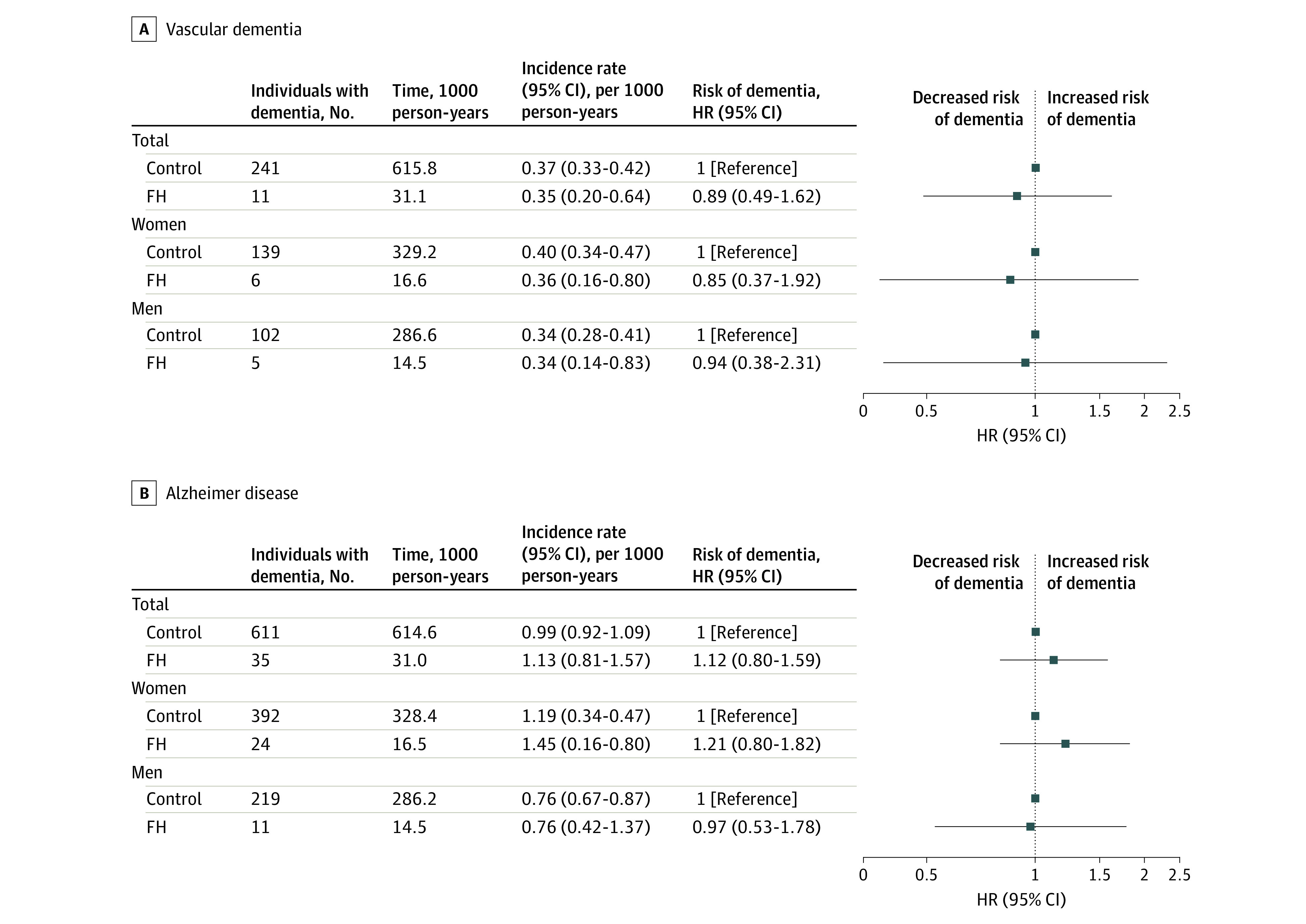
Incidence Rates and Hazard Ratios (HRs) for Vascular Dementia and Alzheimer Disease–Dementia in Patients With Familial Hypercholesterolemia (FH) vs Controls

Among patients with FH vs controls, there was no overall excess risk of total dementia (HR, 0.9; 95% CI, 0.7-1.2) (Table 2), vascular dementia (HR, 0.9; 95% CI, 0.5-1.6) (Figure 1 and eTable 1 in the Supplement), or AD–dementia in AD (HR, 1.1; 95% CI, 0.8-1.6) (Figure 1 and eTable 2 in the Supplement). During the study period, a total of 1356 individuals, including 62 patients with FH (39 women [62.9%]) and 1294 individuals in the control group (801 women [61.9%]) had developed dementia (Table 2). AD–dementia in AD was the most common form of dementia, accounting for 56.5% of all cases (35 patients) among the patients with FH (eTable 2 in Supplement).

**Table 2.** Incidence Rates and HRs for Total Dementia Among Patients With FH vs Controls, 2008-2018

Category	Patients, No.	Person-years, thousands, No.	Incidence rate per 1000 person-years (95% CI)	HR (95% CI)
Total				
Control	1294	613.1	2.11 (2.00-2.23)	1 [Reference]
FH	62	30.9	2.00 (1.56-2.57)	0.93 (0.72-1.20)
Women				
Control	801	327.3	2.45 (2.28-2.62)	1 [Reference]
FH	39	16.5	2.37 (1.73-3.24)	0.95 (0.69-1.30)
Men				
Control	493	285.7	1.73 (1.58-1.88)	1 [Reference]
FH	23	14.5	1.59 (1.06-2.39)	0.90 (0.60-1.37)
Age 40-59 y				
Control	118	449.5	0.26 (0.22-0.31)	1 [Reference]
FH	7	22.7	0.31 (0.15-0.65)	1.17 (0.55-2.51)
Age 60-69 y				
Control	306	110.8	2.76 (2.47-3.09)	1 [Reference]
FH	16	5.5	2.89 (1.88-4.72)	1.04 (0.63-1.72)
Age ≥70 y				
Control	870	52.7	16.5 (15.5-17.6)	1 [Reference]
FH	39	2.7	14.3 (10.5-19.6)	0.86 (0.62-1.19)

Abbreviations: FH, familial hypercholesterolemia; HR, hazard ratio.

**Figure 2.**
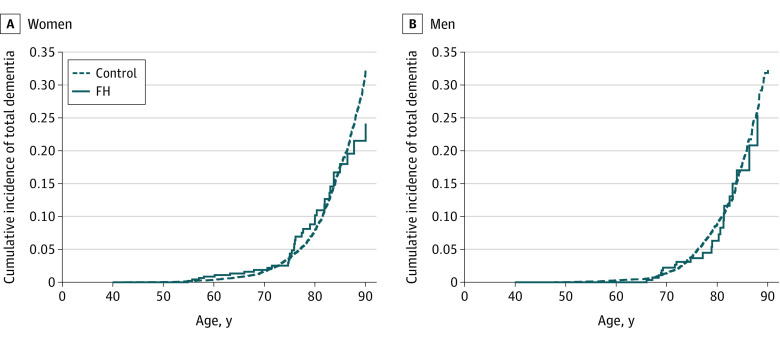
Cumulative Incidence of Total Dementia in the Familial Hypercholesterolemia (FH) and Control Population With Age as the Time Scale

Most dementia cases occurred among patients aged 70 years and older, including 39 patients with FH (62.9%) and 870 patients (67.2%) in the control group (Table 2). The estimated cumulative incidence proportion by age 80 years was 0.10 (95% CI, 0.07-0.15) among women with FH and 0.07 (95% CI, 0.04-0.12) among men with FH (Figure 2). There were no differences in cumulative incidence of total dementia according to age when comparing female patients with FH vs female controls or male patients with FH vs male controls (Figure 2). On the death certificates, there were in total 10 patients with FH and 230 controls with *ICD-10* codes F00-F03 or G30 as the underlying cause of death, and there were 12 patients with FH and 353 controls when including all immediate or contributing causes of death.

### Lipid-Lowering Treatment

Among the 3520 patients with FH, 1795 (50.9%) received a diagnosis in 2004 or later, which gave them a complete follow-up on lipid-lowering drugs from year of FH diagnosis. Among these, 1309 patients (73%) had at least 2 prescriptions of statins (ATC code C10AA) the year of FH diagnosis, 1491 patients (83%) had at least 1 prescription, and 1328 patients (74%) had at least 2 prescriptions of lipid-lowering drugs (ATC code C10A, C10B), mainly statin in combination with ezetimibe. The most common statin used among the 1795 patients with FH was atorvastatin (1471 patients [58.0%]), followed by simvastatin (740 patients [27.5%]), rosuvastatin (253 patients [11.2%]), pravastatin (39 patients [1.5%]), fluvastatin (34 patients [1.3%]), and lovastatin (13 patients [0.5%]). Median daily treatment dose for the first prescription the year of start of follow-up was 40 mg for atorvastatin, simvastatin, rosuvastatin, and lovastatin, and 80 mg for pravastatin and fluvastatin.

There was no association between statin consumption measured as cumulative DDDs of statins and total dementia risk in patients with FH (Table 3). For cumulative DDD of 5000 to 10 000, the HR was 1.2 (95% CI, 0.4-3.8). For the highest cumulative DDD of statins more than 10 000, the HR was 1.9 (95% CI, 0.7-5.0) (Table 3). After further adjustment for comorbidities and comedications, the HR for the highest cumulative dose was reduced to 1.70 (95% CI, 0.57-5.12). The median (IQR) cumulative statin dose until end of follow-up was 8798 (4233-14 795) DDDs in the patients with FH.

**Table 3.** Association Between Cumulative DDDs of Statins and Total Dementia Among 1750 Persons Diagnosed With Familial Hypercholesterolemia From January 1, 2004

Cumulative DDDs	Person-years of follow-up, No.	Patients with dementia, No.	Model 1, HR (95% CI)^a^	*P* value for trend	Model 2, HR (95% CI)^b^	*P* value for trend
1-4999	6672	6	1 [Reference]	.16	1 [Reference]	.26
5000-10 000	4235	6	1.18 (0.37-3.75)		0.75 (0.19-2.89)	
>10 000	3644	13	1.86 (0.69-5.03)		1.70 (0.57-5.12)	

Abbreviations: DDD, defined daily dose; HR, hazard ratio.

^a^ Cox proportional hazards regression was performed with age as the time scale and cumulative dose of statins as time-varying covariate with adjustment for sex.

^b^ Cox proportional hazards regression was performed with age as the time scale and cumulative dose of statins as time-varying covariate with adjustment for sex, previous coronary heart disease, previous hypertension, and use of medications (antithrombotics, antihypertensives, or diabetes treatment) at start of follow-up.

## Discussion

The results of this cohort study showed no excess overall risk of any of the forms of dementia in patients with FH compared with age-matched and sex-matched controls. Significantly more patients with FH were taking statin therapy compared with controls. There was no association between cumulative DDDs of statins, including the highest statin consumption of more than 10 000 DDDs, and total dementia in FH. This result is important because it indicates that long-term use of high-intensity statins is not associated with dementia risk.

Prior studies have shown that early and prolonged statin exposure might prevent dementia,^2,3^ in particular vascular dementia.^22^ The role of statin treatment to prevent AD is, however, controversial.^22^ Once genetically diagnosed, patients with FH receive high-intensity statins.^10^ We have previously reported that 86% of Norwegian patients diagnosed with FH are treated with statins.^23^

A retrospective cohort study^3^ including 6182 individuals with hypercholesterolemia, of whom 1802 were taking statins and 4380 were not, with a median follow-up time 11.7 years, showed a decreased incidence of dementia in those who used statins compared with those who did not. In both sexes, statin exposure decreased dementia risk by approximately 35%.^3^ In that study^3^ the participants had a higher mean age at baseline (66.0 years) compared with our study (51.8 years for patients with FH and 51.7 years for controls at the start of follow-up), and the results were adjusted for several possible confounders.

High levels of midlife cholesterol may be associated with AD risk.^24^ Statins seem beneficial only when uptake begins early in life.^24^ Another study^22^ suggested that statin use may prevent AD and that the prevention may be greater in low-risk individuals. Importantly, in the current study, AD–dementia in AD was the most common form of dementia in the patients with FH, accounting for 56.5% of all cases.

A systematic review^25^ of randomized placebo-controlled trials indicated that statins given late in life to individuals at risk of vascular disease have no effect in preventing AD or dementia. In the present study, the patients with FH had a high mean age (45.1 years) at the time of FH diagnosis, suggesting that the patients were not treated with statins before diagnosis, which may partly explain these findings.

A study^26^ on the incidence of neurodegenerative diseases that was similarly based on *ICD-10* diagnostic codes and used a propensity score–matched population, including 288 515 participants (144 214 patients were exposed to statins and 144 301 were not), showed that statin exposure was associated with a lower incidence of dementia and AD. The mean (SD) follow-up time was 5.1 (2.3) years, and the patients ranged in age from 45 to more than 90 years.^26^ Separate analysis of the incidence of each type of neurodegenerative disease with respect to the different statins was performed.^26^

Importantly, asymptomatic, neurodegenerative processes in the brain may develop several years ahead of dementia. Lowering LDL-C levels by use of statins may slow a conversion from MCI to dementia.^4^ In the current study, we did not include MCI, which may be a prodromal stage for dementia.^27^

Studies on FH and dementia are sparse. One study^27^ on MCI in 47 patients with FH older than 50 years (mean [SD] age, 60 [6.7] years) and 70 matched controls showed a higher incidence of MCI among patients with FH (21.3%) vs controls (2.9%) evaluated by use of neuropsychological tests and brain MRI. These findings were unrelated to white matter disease and were independent of apolipoprotein E4 or E2 status,^27^ suggesting that early exposure to elevated LDL-C levels may be a factor associated with the risk of developing MCI. Another study^4^ recruiting 295 patients with mild MCI older than 50 years aimed to investigate the association of vascular risk factors with AD biomarkers and conversion rate to dementia. Study results showed a positive association between LDL-C levels and plasma levels of biomarkers for AD, including amyloid B and tau proteins, on the basis of multivariable logistic regression models adjusted for age, sex, education, and Mini-Mental State Examination score.^4^

Higher prediagnosis total cholesterol and LDL-C concentrations are associated with faster cognitive decline in patients with incident AD,^6^ suggesting that vascular risk factors play a role in the course of AD. Age is the factor most associated with the risk of developing dementia.^1^ Dementia risk increases with age.^1^ Young-onset dementia (age <65 years) accounts for up to 9% of all cases.^1^ In the present study, approximately 10% of the patients with FH developed total dementia before age 80 years.

Besides age, a healthy lifestyle with healthy diet, no smoking, and regular exercise may prevent dementia.^1^ We have previously shown that individuals with FH have lower levels of smoking-associated cancer, which may indicate this.^28^

### Limitations

This study has limitations that should be considered. The follow-up time was too short (10 years) to reveal any differences in long-term dementia risk. Because the NPR was not established before 2008, diagnostic data on dementia were unavailable for 1992 to 2007, which resulted in left-truncation of follow-up. The lack of follow-up data was, however, the same for both patients with FH and controls, and because the mean age at baseline was young, we do not believe this has introduced any major bias or loss of dementia cases. Another limitation is the lack of information on important confounders, such as lifestyle factors and LDL-C levels. This is important for the analyses of the association between cumulative statin dose and risk of dementia within the FH cohort. Although all persons with FH have an indication for statin use, there could be differences in LDL-C levels between persons with high cumulative doses and persons with lower cumulative dose. The low number of dementia cases among patients with FH may have caused sparse data bias in some of the models, particularly in models for vascular dementia and models for the association between cumulative dose of statins and risk of dementia.

The study results rely entirely on the *ICD-10* code given in the medical records by physicians. Some of the dementia diagnostic codes may have been misclassified because it can be difficult sometimes to give a precise diagnosis before death. Because the diagnosis is clinical, there may also be overlaps between different forms of dementia with similar presentations despite different underlying pathophysiology, which sometimes can be established only by autopsies. Some of the study participants had more than 1 different diagnostic code of dementia in their medical records during follow-up. Some diagnostic codes may have changed during follow-up, or some might have had a mixed form of dementia. Furthermore, some diagnostic codes on dementia were not included in this study, such as Lewy body dementia, frontotemporal dementia, or other forms of neurodegenerative dementias. We had no access to these codes. When ordering data from the NPR, we were obliged to follow strict personal data protection regulations and to restrict the data to the most relevant dementia forms. Furthermore, we only had information on a limited number of covariates. The exact treatment time on statins was lacking as data on pharmaceuticals extracted from the NorPD were not available in Norway before 2004.

## Conclusions

We found no excess risk of total dementia, vascular dementia, or AD–dementia in AD in individuals with genotyped FH compared with age-matched and sex-matched controls in the general Norwegian population during 10 years of follow-up. In addition, there was no association between statin use and dementia risk among individuals with FH.

## Notes

Supplement. **eFigure. **Flow Chart of the Study Population **eTable 1.** Incidence Rates for Vascular Dementia and Hazard Ratios in FH Versus Controls During 2008-2018 **eTable 2.** Incidence Rates for Alzheimer’s Disease/Dementia in Alzheimer’s Disease and Hazard Ratios in FH Versus Controls During 2008-2018
