# Supplementary material for: Association of Familial Hypercholesterolemia and Statin Use With Risk of Dementia in Norway
Source: JAMA Netw Open. 2022 Apr 19;5(4):e227715. doi: 10.1001/jamanetworkopen.2022.7715 (PMC9020214; doi:10.1001/jamanetworkopen.2022.7715)
Supplement: Supplement. — eFigure. Flow Chart of the Study Population eTable 1. Incidence Rates for Vascular Dementia and Hazard Ratios in FH Versus Controls During 2008-2018 eTable 2. Incidence Rates for Alzheimer’s Disease/Dementia in Alzheimer’s Disease and Hazard Ratios in FH Versus Controls During 2008-2018 [file jamanetwopen-e227715-s001.pdf]

Supplemental Online Content

Mundal LJ, Igland J, Svendsen K, Holven KB, Leren TP, Retterstøl K. Association of familial hypercholesterolemia and statin use with risk of dementia in Norway. *JAMA Netw Open*. 2022;5(4):e227715. doi:10.1001/jamanetworkopen.2022.7715

eFigure. Flow Chart of the Study Population
eTable 1. Incidence Rates for Vascular Dementia and Hazard Ratios in FH Versus Controls During 2008-2018
eTable 2. Incidence Rates for Alzheimer's Disease/Dementia in Alzheimer's Disease and Hazard Ratios in FH Versus Controls During 2008-2018

This supplemental material has been provided by the authors to give readers additional information about their work.

© 2022 Mundal LJ et al. *JAMA Network Open.*

eFigure. Flow Chart of the Study Population

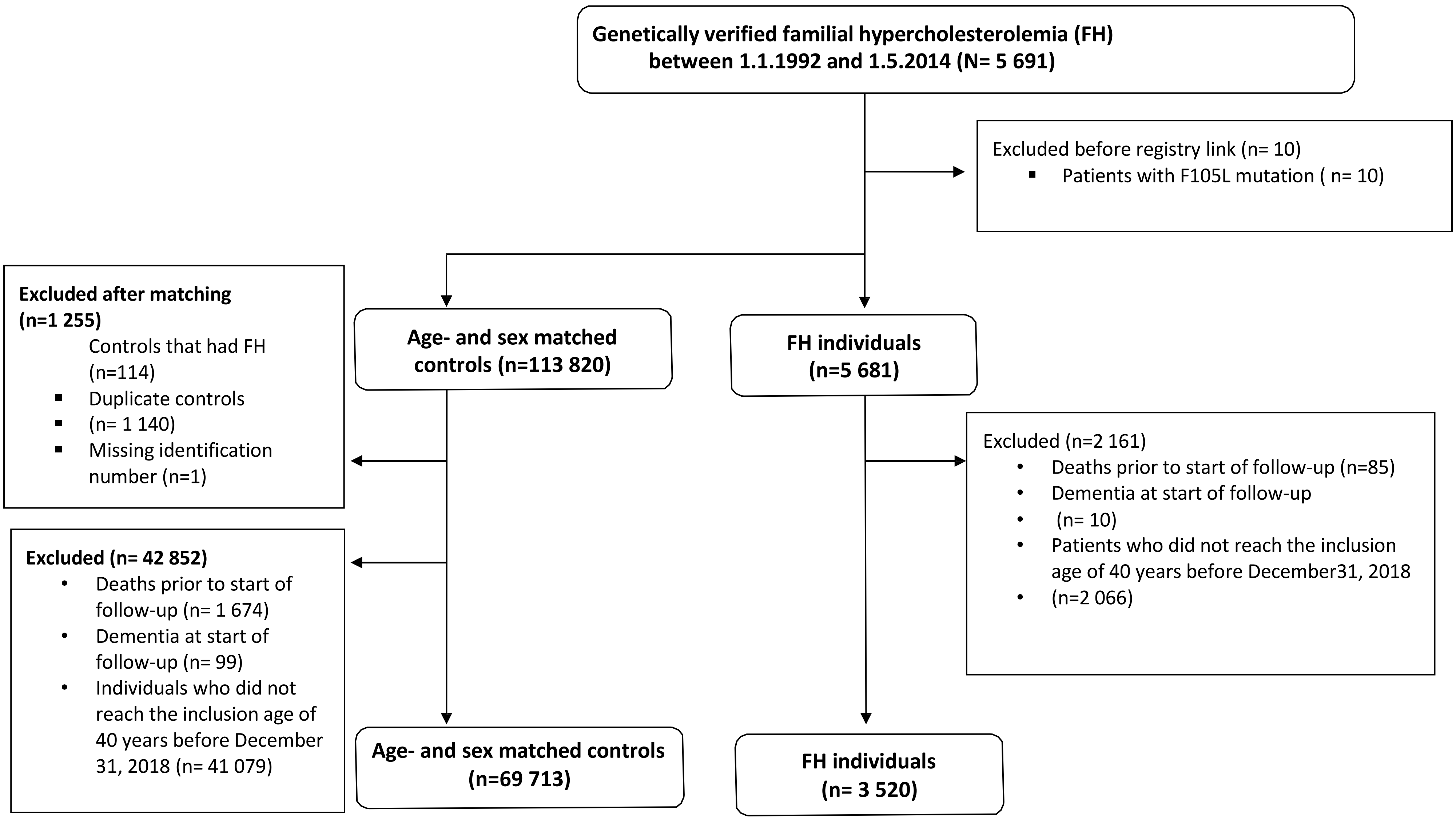

© 2022 Mundal LJ et al. *JAMA Network Open.*

eTable 1. Incidence Rates for Vascular Dementia and Hazard Ratios in FH Versus Controls During 2008-2018

		N	Person years in 1000	Incidence rate per 1000 Person years (95% CI)	HR (95% CI)
Total					
	Control	241	615.8	0.37 (0.33-0.42)	1
	FH	11	31.1	0.35 (0.20-0.64)	0.89 (0.49-1.62)
Women					
	Control	139	329.2	0.40 (0.34-0.47)	1
	FH	6	16.6	0.36 (0.16-0.80)	0.85 (0.37-1.92)
Men					
	Control	102	286.6	0.34 (0.28-0.41)	1
	FH	5	14.5	0.34 (0.14-0.83)	0.94 (0.38-2.31)

N= number of patients, 95 % CI: 95% confidence interval.
HR: hazard ratio. FH: familial hypercholesterolemia

© 2022 Mundal LJ et al. *JAMA Network Open.*

eTable 2. Incidence Rates for Alzheimer's Disease/Dementia in Alzheimer's Disease and Hazard Ratios in FH Versus Controls During 2008-2018

		N	Person years in 1000	Incidence rate per 1000 Person years (95% CI)	HR (95% CI)
Total					
	Control	611	614.6	0.99 (0.92-1.09)	1
	FH	35	31.0	1.13 (0.81-1.57)	1.12 (0.80-1.58)
Women					
	Control	392	328.4	1.19 (1.08-1.32)	1
	FH	24	16.5	1.45 (0.97-2.17)	1.21 (0.80-1.82)
Men					
	Control	219	286.2	0.76 (0.67-0.87)	1
	FH	11	14.5	0.76 (0.42-1.37)	0.97 (0.53-1.78)

N= number of patients, 95 % CI: 95% confidence interval.
HR: hazard ratio. FH: familial hypercholesterolemia

© 2022 Mundal LJ et al. *JAMA Network Open.*
